# A cross‐sectional and prospective examination of alcohol use and misuse among adult twin and sibling pairs discordant for neighborhood socio‐economic disadvantage

**DOI:** 10.1111/add.70533

**Published:** 2026-06-29

**Authors:** Wendy S. Slutske, Timothy B. Baker, Thomas M. Piasecki

**Affiliations:** ^1^ Department of Family Medicine and Community Health, School of Medicine and Public Health University of Wisconsin‐Madison Madison WI USA; ^2^ Center for Tobacco Research and Intervention, School of Medicine and Public Health University of Wisconsin‐Madison Madison WI USA; ^3^ Division of General Internal Medicine, Department of Medicine, School of Medicine and Public Health University of Wisconsin‐Madison Madison WI USA

**Keywords:** Add Health, alcohol quantity × frequency, alcohol use, discordant twins and siblings, neighborhood disadvantage, prospective, quasi‐experimental

## Abstract

**Aim:**

To determine whether the association between neighborhood disadvantage and alcohol use and misuse can be explained by a potentially causal effect of neighborhoods or by between‐family genetic and environmental confounding.

**Design:**

Cross‐sectional and prospective discordant twin and sibling study.

**Setting:**

United States national sample.

**Participants:**

1477 adult twin and sibling pairs (217 monozygotic twin, 336 dizygotic twin and 924 full biological sibling pairs) from the National Longitudinal Study of Adolescent to Adult Health (mean age = 28.49 years, 53% female). Most twin and sibling pairs were discordant for the level of disadvantage in their neighborhood (70%).

**Measurements:**

The predictor was wave 4 neighborhood disadvantage, along with wave 4 individual‐, family‐ and neighborhood‐level covariates. The outcomes were any alcohol use, quantity x frequency of alcohol use (alcohol QF) and frequency of binge drinking assessed at wave 4 in the cross‐sectional models and at wave 5 in the prospective models. Neighborhood disadvantage for the two members of a twin/sibling pair was used to create two orthogonal within‐ and between‐pair variables to be used to decompose the neighborhood/alcohol association into a within‐pair (i.e. potentially causal) effect and a between‐pair association (i.e. family level confounding).

**Findings:**

In fully adjusted models including all covariates, only between‐pair (not within‐pair) neighborhood disadvantage was cross‐sectionally [odds ratio (OR) = 0.92, 95% confidence interval (CI) = 0.88–0.98] and prospectively (OR = 0.92, 95% CI = 0.85–0.98) associated with lower odds of any alcohol use in the past year. Between‐pair neighborhood disadvantage was cross‐sectionally, but not prospectively, associated with increased alcohol QF in the past month among women [mean ratio (MR) = 1.09, 95% CI = 1.03–1.15], but not among men (MR = 1.01, 95% CI = 0.96–1.06). Within‐pair disadvantage was also cross‐sectionally, but not prospectively, associated with increased alcohol QF in the past month (MR = 1.04, 95% CI = 1.01–1.07). Neighborhood disadvantage was not significantly associated with binge drinking.

**Conclusions:**

Neighborhood disadvantage appears to be negatively, positively and not significantly associated with any alcohol use, quantity‐frequency of use and binge drinking, respectively. Neighborhood disadvantage associations are more consistently explained by between‐family confounds, that is, genetic and family environmental factors common to neighborhood disadvantage and to drinking occurrence and amount consumed. This may be due to differential selection by families into neighborhoods.

## INTRODUCTION

Where one lives is associated with disparities in health behaviors and outcomes [1, 2, 3], but it has been difficult to draw firm conclusions about the source of the relations between neighborhood socio‐economic disadvantage (i.e. living in a neighborhood in which many of the residents are living in poverty, receive public assistance or are unemployed) and alcohol use and misuse [4]. It is usually assumed that neighborhoods are exogenous to the individual [5, 6], despite robust evidence that family [7] and genetic differences [8, 9] are associated with the nature of the neighborhood in which one lives. Studies of exposure‐discordant twins can examine the relations between neighborhood factors and alcohol use and misuse controlling for these between‐family differences, that is, controlling for genetic and shared environmental confounding with neighborhood [10, 11, 12, 13, 14].

However, to our knowledge, only two studies have used this approach to investigate the relation between neighborhood disadvantage and alcohol use or misuse. Among twin pairs from the Washington State Twin Registry (mean age = 36.6 years, 66% female), there was a significant positive within‐twin‐pair association and a significant negative between‐twin‐pair association between neighborhood deprivation and hazardous drinking after accounting for sex, race, income, educational attainment, rurality and alcohol outlet density [15]. The significant positive within‐pair association indicates that the twin living in a more deprived neighborhood engaged in more hazardous drinking than his or her co‐twin living in a less deprived neighborhood, whereas the significant negative between‐twin‐pair association indicated that individuals from pairs with higher average neighborhood deprivation engaged in less hazardous drinking than those from pairs with lower average neighborhood deprivation. These seemingly anomalous findings suggest that neighborhood deprivation may potentially cause 1 *more* hazardous drinking coupled with familial factors resulting in an association of neighborhood deprivation with *less* hazardous drinking. Among twin pairs from the Australian Twin Registry (mean age = 37.7 years, 57% female), there was no evidence for a within‐twin‐pair association between neighborhood disadvantage and past‐year alcohol use disorder symptoms, but (similar to [15]) there was a significant negative between‐twin‐pair association between neighborhood disadvantage and past‐year alcohol use disorder symptoms [16]. That is, after accounting for sex, age, household income, educational attainment and personality dimensions, twins from pairs who, on average, were living in less disadvantaged neighborhoods reported more past‐year alcohol use disorder symptoms than those living, on average, in more disadvantaged neighborhoods [16].

The two previous studies provided mixed evidence about whether there is a potentially causal within‐twin‐pair effect of neighborhood disadvantage on alcohol misuse but were consistent in demonstrating a significant inverse relation of between‐twin‐pair neighborhood disadvantage and alcohol misuse that survived covariate controls. However, the previous studies had some limitations. First, both the Washington State and Australian twin registries included very few non‐White individuals. Second, none of the covariates in these studies targeted the between‐family effects observed. Third, both studies were cross‐sectional and therefore could not establish the temporal precedence of neighborhood disadvantage.

The present study was guided by ecological models of health behavior, which emphasize the multiple levels of influence on behaviors, from the intrapersonal (individual), to the interpersonal (family), to the community (neighborhood) [17]. The relations between neighborhood disadvantage and alcohol use and misuse in a sample of 1477 twin and sibling pairs identified from the nationally representative 2 (and racially and ethnically diverse) National Longitudinal Study of Adolescent to Adult Health (Add Health [18, 19]) were dissected into within‐pair and between‐pair influences before and after controlling for individual‐, family‐ and neighborhood‐level influences. Many of the same individual‐level covariates, that is, income, educational attainment [15, 16] and personality traits [16], and neighborhood‐level covariates, that is, rural residence [15], as used in the previous studies were included. Such characteristics might explain any observed within‐pair effects. We also included parent‐reported family socio‐economic status (SES) assessed when the participants were adolescents, a characteristic shared by twins and siblings that might inform any between‐pair effects [7]. Unlike the previous studies, we were also able to establish temporal precedence by predicting alcohol use and misuse nearly a decade after exposure to living in a disadvantaged neighborhood.

## METHODS

### Participants

Participants were drawn from Add Health, a longitudinal study of a nationally representative sample of adolescents, born between March 1974 and July 1983, who were in grades 7–12 in the USA in 1994–1995 [18, 19]. Variables of interest for the current study (except parental SES) were obtained in wave 4 (*n* = 15 701), which was conducted in 2008–2009 with a response rate of 80.3%. Alcohol outcomes at wave 5 (*n* = 12 300), which was conducted in 2016–2018 with a response rate of 71.8%, were also included for prospective analyses.

Among the 15 701 participants in wave 4 were 2954 individuals from 1477 complete twin or full sibling pairs. This included 217 monozygotic twin (MZ), 336 dizygotic twin (DZ) and 924 full biological sibling (FS) pairs; the mean age of the participants at wave 4 was 28.44 years (range = 24–32 years, SE = 0.04 years) and 52% were female. These were the focus of the present study. The twin and FS participants came from 49 different states and from 2180 different census tracts [20]. 3 Of the 2954 individuals in wave 4 from complete twin/sibling pairs, 2046 (69%) participated in wave 5 (mean age = 37.55 years, 56% female). The mean interval between waves 4 and 5 for this subsample was 9.09 years (SD = 7.02 years) (for more details, see Table 1).

**TABLE 1:** Description of participants at wave 4 and wave 5.

	Wave 4	Wave 5
Years of data collection	2008–2009	2016–2018
Sample size	2954 individuals	2046 individuals
Characteristic	% (*n*) or mean (SE)	% (*n*) or mean (SE)
Sex		
Male	48 (1412)	44 (900)
Female	52 (1542)	56 (1146)
Age	28.44 (0.04)	37.55 (0.04)
Race/ethnicity^a^		
White	58 (1700)	62 (1253)
Black/African American	14 (399)	13 (256)
Hispanic/Latino	20 (584)	18 (368)
Asian/Pacific Islander	7 (193)	6 (123)
Native American	2 (52)	2 (32)
Twin/sibling type		
Monozygotic twin	15 (434)	14 (294)
Dizygotic twin	23 (672)	21 (436)
Full sibling^b^	62 (1848)	64 (1316)
Level of discordance^c^		
0 deciles, concordant	30 (864)	29 (584)
1 decile	19 (550)	20 (398)
2 deciles	15 (426)	14 (292)
3 deciles	12 (346)	12 (237)
4 deciles	8 (224	7 (146)
5+ deciles	17 (506)	18 (363)

*Note*: Individuals come from 1477 complete twin/sibling pairs in wave 4.

^a^ Missing race at wave 4 (*n* = 26) and wave 5 (*n* = 14).

^b^ The mean age difference for full siblings was 2.28 years (SD = 1.16).

^c^ Concordance/discordance for level of neighborhood disadvantage; missing discordance at wave 4 (*n* = 38) and wave 5 (*n* = 26).

### Measures

Table 2 lists the measures and the Add Health wave when they were assessed.

**TABLE 2:** Correlations between study variables.

Wave 4 variables (except parental SES)	Wave 4			Wave 5		Mean	SE
	ND	Any use	Alc Q × F	Any use	Alc Q × F		
Neighborhood disadvantage	–	**−0.14**	**0.11**	**−0.13**	−0.01	4.41	0.06
Age	**−0.03**	**−0.05**	0.01	−0.01	−0.02	28.44	0.04
Race/ethnicity^a^	**−0.25**	**0.15**	0.01	**0.08**	**0.09**	0.58	0.01
Sex^b^	0.00	**−0.12**	**−0.19**	0.01	**−0.18**	1.52	0.01
Rural	**0.18**	**−0.08**	0.01	−0.05	0.06	0.10	0.01
Parental SES^c^ (wave 1)	**−0.30**	**0.17**	−0.02	**0.17**	**0.06**	0.05	0.03
Household income^d^	**−0.29**	**0.17**	**−0.05**	**0.17**	0.01	6.30	0.08
Educational attainment^e^	**−0.24**	**0.17**	**−0.13**	**0.12**	**−0.06**	2.75	0.03
Neuroticism^c^	**0.10**	**−0.07**	0.05	−0.04	0.01	0.01	0.02
Extraversion^c^	**−0.06**	**0.13**	**0.09**	**0.09**	**0.07**	0.01	0.02
Openness^c^	**−0.05**	**0.14**	0.01	**0.06**	−0.01	0.00	0.02
Agreeableness^c^	**−0.10**	**0.08**	**−0.12**	**0.08**	**−0.08**	0.00	0.02
Conscientiousness^c^	**−0.06**	−0.02	−0.04	−0.03	0.00	0.00	0.02
Mean	4.41	0.70	26.68	0.84	27.58	–	–
Standard error of mean	0.06	0.01	1.16	0.01	1.34	–	–

*Note*: Estimates in bold are significant at *P* < 0.05.

Abbreviations: Alc Q × F = past‐month quantity × frequency of alcohol use; Any use = any past‐year alcohol use; ND = neighborhood disadvantage; SES = socio‐economic status.

^a^ Race/ethnicity coding: 1 = White; 0 = non‐White (only for this table).

^b^ Sex coding: 1 = male; 2 = female.

^c^ Variables have been converted to *z*‐scores (mean = 0, SD = 1).

^d^ Household income is divided by 10 000.

^e^ Educational attainment was coded into a six‐level ordinal variable with the following categories: 1 = less than high school; 2 = high school graduate; 3 = completed vocational or technical training; 4 = college degree; 5 = master's degree; 6 = PhD, MD or other advanced degree.

#### Alcohol use and misuse

Two alcohol use outcomes were examined: any alcohol use in the past year and quantity × frequency of alcohol use in the past month. The two alcohol outcomes were complementary in that any alcohol use is an indicator of *whether* the participant drank and alcohol quantity × frequency is an indicator of, among those who drank, *how much* they drank. In addition, one alcohol misuse outcome was examined: frequency of binge drinking in the past year. All outcomes were assessed at waves 4 and 5.

Past‐month quantity × frequency of alcohol use was the product of quantity and frequency of alcohol use. Past‐month quantity of use was assessed with the question ‘Think of all the times you have had a drink during the past 30 days. How many drinks did you usually have each time? A “drink” is a glass of wine, a can or bottle of beer, a wine cooler, a shot glass of liquor or a mixed drink’, with open‐ended responses. This variable was winsorized [21] at 15 drinks per occasion. Past‐month frequency was assessed with the question ‘During the past 30 days, on how many days did you drink alcohol (beer, wine or liquor)?’, with seven response categories ranging from ‘none’ to ‘every day or almost every day’. These categories were used to create a continuous frequency measure by using the midpoint of the category bin. Past‐year binge drinking was assessed with the question ‘During the past 12 months, on how many days did you drink (female = 4/male = 5) or more drinks in a row?’ with seven response categories ranging from ‘none’ to ‘every day or almost every day’. These categories were used to create a continuous frequency of binge drinking measure by using the midpoint of the category bin.

#### Neighborhood socio‐economic disadvantage

Participants’ addresses in 2007–2008 at wave 4 were linked with census tract‐level data from the 2005–2009 panels of the American Community Survey [22, 23]. A measure of neighborhood socio‐economic disadvantage was created based on the following five indicators extracted from census data for each census tract: proportions of female single‐parent households; individuals living below the poverty threshold; individuals receiving public assistance; adults with less than a high school education; and adults who were unemployed. Each of the indicators was converted to deciles and these were summed to create the neighborhood disadvantage composite measure. 4 The measure of neighborhood disadvantage was a continuous measure ranging from 5 to 50; this was used to create neighborhood disadvantage deciles ranging from 0 to 9. (More information about the neighborhood socio‐economic disadvantage measure is available in Table S1.)

#### Rural residence

Rural–urban commuting area (RUCA) codes were based on measures of population density, urbanization and daily commuting from the 2000 decennial census [24]. RUCA codes 1–6 were classified as ‘urban’, and codes 7–10 were classified as ‘rural’.

#### Parental socio‐economic status

Parental SES was based on parent reports obtained from Add Health wave 1 when the participants were adolescents, and included parental education, parental occupation, household income and household receipt of public assistance [25].

#### Personal socio‐economic status

Two indicators of personal SES included past‐year household income and educational attainment. Educational attainment was coded into a six‐level ordinal variable with the following categories: 1 = less than high school; 2 = high school graduate; 3 = completed vocational or technical training; 4 = college degree; 5 = master's degree; and 6 = PhD, MD or other advanced degree.

#### Personality traits

Participants completed the Mini‐IPIP (International Personality Item Pool), a 20‐item inventory designed to assess the Big Five factors of personality [26]. For each item, individuals were asked ‘How much do you agree with each statement about you as you generally are now, not as you wish to be in the future?’; responses were given using a five‐point Likert‐type scale, ranging from 1 (strongly agree) to 5 (strongly disagree). Each of the Big Five factors (neuroticism, extraversion, openness, agreeableness and conscientiousness) was assessed via four items. An individual's scores for each factor were computed by summing across factor‐specific items and then standardizing the variables into *z*‐scores (mean = 0, SD = 1).

### Analytic plan

#### Descriptive analyses

Associations between study variables were estimated using a survey data analysis procedure in SAS 9.4 [27] that accounted for the non‐independence of twin/sibling‐pair observations.

#### Multi‐level modeling analyses

The estimation of multi‐level models was conducted using PROC GLIMMIX for generalized linear mixed models (GLMMs) in SAS. GLMM is a form of multi‐level modeling for the analysis of clustered data with non‐normal dependent measures [28]. Logistic regression was used to predict any alcohol use. As appropriate for a highly skewed consumption variable that is constrained to positive values [29], gamma regression was used to predict alcohol quantity × frequency. Negative binomial regression was used to predict frequency of binge drinking. Two sets of models were fitted with alcohol use and misuse at waves 4 (cross‐sectional) and 5 (prospective) as the outcomes. Models were first run at the individual level that accounted for the non‐independence of twin/sibling‐pair observations. These analyses examined evidence for an overall association between neighborhood disadvantage and alcohol use and misuse. Next, discordant twin/sibling models were fitted to remove potential sources of confounding that may contribute to the overall association observed in individual‐level models [11]. Compared with the analysis of unrelated individuals, discordant twin/sibling modeling controls for shared family environmental and genetic factors, which makes this method uniquely suited to parsing familial effects from possible causal effects of an environmental exposure variable.

The general form of the discordant twin/sibling model (using any alcohol use as an example) is AU = β_0_ + β_b_ (mean pair ND) + β_w_ (individual ND – mean pair ND) + error, where AU = presence or absence of any alcohol use for each twin/sibling and ND = neighborhood disadvantage for each twin/sibling. The coefficient β_b_ refers to the between‐pair effect and represents variation accounted for by familial factors shared by both twin/siblings in a pair. The coefficient β_w_ represents the within‐pair (i.e. potentially causal) effect, or the difference in the odds of any alcohol use between the twin/sibling living in a neighborhood higher in disadvantage and the twin/sibling living in a neighborhood lower in disadvantage. These two results are not mutually exclusive, and neighborhood disadvantage could have both a potentially causal effect on any alcohol use as well as be associated with any alcohol use via between‐family differences.

The multi‐level models included the control variables of sex (coded 1 for male, 2 for female), age and race/ethnicity (dummy‐coded White, Black or other). Two control variables were evaluated and only retained in models when statistically significant: (i) whether the DZ (dizygotic) or FS (full sibling) pairs were the same or unlike sex (coded 1 for female/female and male/male pairs, and 0 for female/male pairs); and (ii) sibling pair genetic dissimilarity, for brevity, ‘zygosity’ (coded 0 for MZ pairs and 1 for DZ and FS pairs); sensitivity analyses comparing the correlations in FS and DZ pairs showed similar levels of association for the two twin/sibling types (see Appendix S1). The interactions between zygosity and within‐pair neighborhood disadvantage, 5 and between both sex and race/ethnicity with within‐pair and between‐pair neighborhood disadvantage, were also evaluated and retained in models when statistically significant.

Because comparing discordant twins and siblings only controls for genetic and shared environmental background factors, there still exists the possibility that individual‐specific factors related to twin/sibling discordance might confound the within‐pair effects. Similarly, there may be family‐wide factors related to differences between twin/sibling pairs from the same family that might confound (and potentially explain) the between‐pair effects.

Fully adjusted models included the effects of: (i) rural residence; (ii) parental SES; (iii) personal SES (household income and educational attainment); and (iv) the Big Five personality traits. Of particular interest was the significance of the covariate effects, as well as the extent to which the inclusion of the individual‐, family‐ and neighborhood‐level covariates reduced or eliminated the within‐ and between‐pair effects of neighborhood disadvantage. Sampling weights were not constructed for the Add Health genetic supplemental sample [30].^2^ The analysis plans were not pre‐registered and therefore the results should be considered exploratory.

## RESULTS

### Sample characteristics

Most twin/sibling pairs lived in different census tracts (83%) and were discordant for their level of neighborhood disadvantage (70%) at wave 4 (see Table 1). The average twin/sibling pair discordance was 2.20 (SD = 2.22) deciles. Seventy‐five percent of the twins or siblings had moved in the 9 years between Add Health waves 4 and 5. The mean distance moved was 151 miles (SD = 455 miles, range = 0.26–5614 miles) and 69% had moved to a different census tract (based on the 94% with complete geocoding at both time points). 6

### Correlations between study variables

The cross‐sectional and prospective associations between neighborhood disadvantage and any alcohol use were significant and negative; the cross‐sectional association with alcohol quantity × frequency was significant and positive but the prospective association was not significant (see Table 2). The cross‐sectional and prospective associations between neighborhood disadvantage and binge drinking were not significant and therefore were not interrogated further (Table S1 shows the relation between neighborhood disadvantage and the three alcohol outcomes as a function of time lag and wave across five waves of the Add Health study).

Parental SES, household income, educational attainment and the personality traits of neuroticism, extraversion, openness and agreeableness were significantly associated with both any alcohol use at waves 4 and 5 and neighborhood disadvantage; educational attainment and extraversion and agreeableness were also associated with alcohol quantity × frequency at waves 4 and 5 (Table 2). This implicates these covariates as potentially accounting for any observed within‐pair (i.e. potentially causal) or between‐pair (i.e. family‐level confounding) effects of disadvantage on any alcohol use or quantity × frequency of use in the multi‐level discordant twin/sibling models.

### Multi‐level models of any past‐year alcohol use

#### Individual‐level models

In minimally adjusted models that co‐varied sex, age and race/ethnicity, there were significant inverse cross‐sectional and prospective associations between neighborhood disadvantage and any alcohol use at wave 4 (OR = 0.90, 95% CI = 0.87–0.93) and wave 5 (OR = 0.88, 95% CI = 0.84–0.93), respectively. Discordant twin/sibling models can clarify whether these associations were associated with a potentially causal effect of neighborhood disadvantage on alcohol use or between‐family confounding.

#### Cross‐sectional discordant twin/sibling models

**FIGURE 1:**
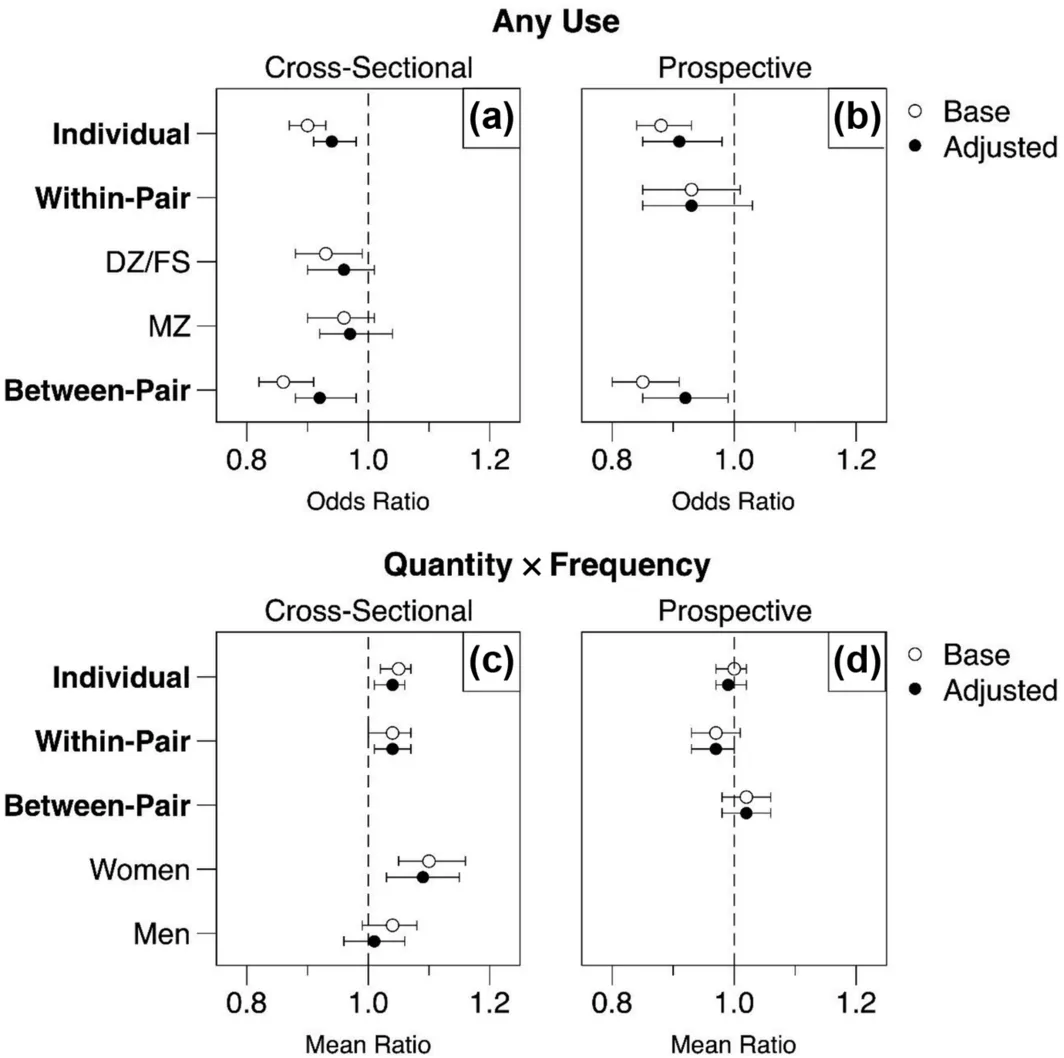
Estimates (and 95% confidence intervals) from individual‐level and multi‐level discordant twin/sibling models cross‐sectionally and prospectively predicting past‐year any alcohol use and past‐month quantity × frequency of use from neighborhood socio‐economic disadvantage. In each panel, individual‐level effects are in the top row, within‐pair (potentially causal) effects are in the middle row and between‐pair effects (family‐level confounding) are in the bottom row. Because there was a significant interaction between zygosity and the within‐pair effect for any alcohol use, separate estimates are provided for DZ/FS and MZ pairs (a). Similarly, separate estimates are provided for women and men because there was a significant interaction between sex and the between‐pair effect for alcohol quantity × frequency (c). The base models included age, sex and race/ethnicity as covariates, and the adjusted models also included rural residence, parental socio‐economic status (in adolescence), household income, educational attainment and the Big Five personality traits as covariates. DZ/FS = dizygotic twin/full sibling pairs; MZ = monozygotic twin pairs.

In the base model, the within‐pair association was qualified by a significant zygosity interaction wherein the association was significant among DZ/FS pairs but not among MZ pairs, but this interaction was no longer significant in the full model (Figure 1a; Table 3). There was also a significant between‐pair association between neighborhood disadvantage and alcohol use (Table 3). Each additional between‐pair decile in neighborhood disadvantage was associated with a 14% decreased odds of alcohol use (Table 3). In other words, the odds of alcohol use was higher in more *advantaged* than in more *disadvantaged* neighborhoods (the between‐pair association persisted even after accounting for wave 3 alcohol use; see Table S2). The between‐pair association between neighborhood disadvantage and alcohol use in the full model simultaneously including all the individual‐, family‐ and neighborhood‐level covariates was reduced, but remained statistically significant (Figure 1a; Table 3).

**TABLE 3:** Results from cross‐sectional multi‐level discordant twin/sibling models predicting any past‐year use and past‐month quantity × frequency of alcohol use at wave 4 from neighborhood disadvantage at wave 4.

Predictor	Past‐year any alcohol use^b^				Past‐month quantity × frequency of use^c^			
	Base model		Fully adjusted model		Base model		Fully adjusted model	
	OR	95% CI	OR	95% CI	MR	95% CI	MR	95% CI
Within‐pair ND	1.09	0.95–1.26	1.11	0.95–1.30	1.04	1.00–1.07	**1.04**	**1.00–1.07** ^f^
Between‐pair ND	**0.86**	**0.82–0.91**	**0.92**	**0.88–0.98**	1.03	0.99–1.08	1.02	0.97–1.06
Zygosity^a^	1.21	0.90–1.62	**1.51**	**1.11–2.07**	–	–	–	–
Zygosity × within‐pair ND^d^	**0.85**	**0.73–1.00**	0.85	0.72–1.01	–	–	–	–
Sex × between‐pair ND^e^	–	–	–	–	**1.07**	**1.01–1.13**	**1.07**	**1.01–1.14**
Female sex	**0.52**	**0.43–0.64**	**0.54**	**0.43–0.69**	**0.34**	**0.26–0.45**	**0.35**	**0.26–0.45**
Age	**0.92**	**0.87–0.97**	**0.91**	**0.86–0.97**	0.97	0.93–1.01	0.98	0.95–1.02
Race/ethnicity								
White (reference)	–	–	–	–	–	–	–	–
Black	**0.51**	**0.38–0.68**	**0.57**	**0.42–0.78**	**0.70**	**0.56–0.86**	**0.78**	**0.63–0.98**
Other	**0.61**	**0.47–0.79**	**0.59**	**0.44–0.79**	0.97	0.81–1.16	0.94	0.78–1.14
Rural			**0.57**	**0.40–0.82**			0.97	0.74–1.26
Parental SES			1.05	0.95–1.15			1.07	1.00–1.14
Household income			**1.07**	**1.04–1.11**			1.00	0.98–1.01
Educ. attainment			**1.29**	**1.16–1.44**			0.97	0.91–1.03
Personality								
Neuroticism			1.00	0.90–1.12			**1.16**	**1.09–1.23**
Extraversion			**1.30**	**1.16–1.46**			**1.24**	**1.16–1.32**
Openness			**1.15**	**1.02–1.29**			1.01	0.94–1.08
Agreeableness			1.07	0.95–1.21			**0.86**	**0.79–0.92**
Conscientiousness			**0.82**	**0.73–0.91**			1.00	0.94–1.07

*Note*: Estimates in bold are significant at *P* < 0.05.

Abbreviations: CI = confidence interval; DZ = dizygotic; FS = full sibling; MR = mean ratio; MZ = monozygotic; ND = neighborhood disadvantage; OR = odds ratio; SES = socio‐economic status.

^a^ Zygosity is coded ‘0’ for MZ pairs and ‘1’ for DZ/FS pairs.

^b^ Main effects for same‐sex sibling and interactions between sex and race and the within‐pair and/or between‐pair neighborhood disadvantage effects were not significant and were not included in the models.

^c^ Main effects for same‐sex sibling and zygosity and the interactions between zygosity and race and the within‐pair and/or between‐pair neighborhood disadvantage effects were not significant and were not included in the models.

^d^ Difference in base model: DZ/FS, OR = 0.93 (95% CI = 0.88–0.99); MZ, OR = 0.96 (95% CI = 0.90–1.01). No difference in full model.

^e^ Difference in base model: females, MR = 1.10 (95% CI = 1.05–1.16); males, MR = 1.04 (95% CI = 0.99–1.08). Difference in full model: females, MR = 1.09 (95% CI = 1.03–1.15); males, MR = 1.01 (95% CI = 0.96–1.06).

^f^ *P* = 0.03.

#### Prospective discordant twin/sibling models

The results of the prospective models predicting alcohol use at wave 5 from neighborhood disadvantage at wave 4 were similar to the results of the cross‐sectional models. In the base prospective model, there was a significant between‐pair association, but not within‐pair association, between neighborhood disadvantage and any alcohol use (Figure 1b; Table 4). Each additional between‐pair decile in neighborhood disadvantage was associated with a 15% decreased odds of alcohol use 9 years later (the between‐pair association persisted even after accounting for wave 4 alcohol use and wave 5 neighborhood disadvantage; see Tables S3 and S4). In the full model simultaneously including all the individual‐, family‐ and neighborhood‐level covariates, the prospective between‐pair association between neighborhood disadvantage and alcohol use was substantially reduced but remained statistically significant (Figure 1b; Table 4). Prior to the final model fitting, a control variable and several interactions were evaluated, and as they were not significantly associated with any alcohol use, they were not included in the models; see Tables 3 and 4 and Appendix S1.

**TABLE 4:** Results from prospective multi‐level discordant twin/sibling models predicting any past‐year and past‐month quantity × frequency of alcohol use at wave 5 from neighborhood disadvantage at wave 4.

Predictor	Past‐year any alcohol use^b^				Past month quantity × frequency of use^c^			
	Base model		Fully adjusted model		Base model		Fully adjusted model	
	OR	95% CI	OR	95% CI	MR	95% CI	MR	95% CI
Within‐pair ND	0.93	0.85–1.01	0.93	0.85–1.03	0.97	0.93–1.01	0.97	0.93–1.00
Between‐pair ND	**0.85**	**0.80–0.91**	**0.92**	**0.85–1.00** ^d^	1.02	0.98–1.06	1.02	0.98–1.06
Female sex	0.96	0.72–1.29	0.91	0.64–1.30	**0.54**	**0.46–0.63**	**0.57**	**0.48–0.67**
Age	0.96	0.88–1.04	0.93	0.84–1.02	0.97	0.93–1.01	**0.95**	**0.91–0.99**
Race/ethnicity								
White (reference)	–	–	–	–	–	–	–	–
Black	0.68	0.46–1.01	0.81	0.51–1.29	**0.73**	**0.58–0.92**	0.82	0.64–1.05
Other	0.89	0.61–1.31	1.04	0.66–1.62	**0.68**	**0.55–0.84**	**0.79**	**0.56–0.86**
Rural			0.96	0.57–1.63			1.23	0.91–1.65
Parental SES			**1.26**	**1.09–1.46**			**1.17**	**1.08–1.26**
Household income			**1.10**	**1.05–1.16**			1.01	0.99–1.03
Educational attainment			1.08	0.93–1.27			0.96	0.90–1.03
Personality								
Neuroticism			1.07	0.90–1.26			**1.09**	**1.01–1.17**
Extraversion			**1.37**	**1.15–1.63**			**1.23**	**1.14–1.33**
Openness			1.03	0.87–1.23			**0.92**	**0.85–0.99**
Agreeableness			1.10	0.92–1.33			**0.89**	**0.81–0.97**
Conscientiousness			**0.80**	**0.68–0.95**			1.07	0.99–1.15

*Note*: Estimates in bold are significant at *p* < 0.05.

Abbreviations: CI = confidence interval; MR = mean ratio; ND = neighborhood disadvantage; OR = odds ratio; SES = socio‐economic status.

^b^ Main effects for same‐sex sibling and zygosity and the interactions between zygosity, sex and race and the within‐pair and/or between‐pair neighborhood disadvantage effects were not significant and were not included in the models.

^c^ Main effects for same‐sex sibling and zygosity and the interactions between zygosity, sex and race and the within‐pair and/or between‐pair neighborhood disadvantage effects were not significant and were not included in the models.

^d^ *P* = 0.04.

### Multi‐level models for past‐month quantity × frequency of alcohol use

#### Individual‐level models

In minimally adjusted models that co‐varied sex, age and race/ethnicity, there was a significant cross‐sectional association at wave 4 [mean ratio, MR (exponentiated gamma regression coefficient) = 1.05, 95% CI = 1.02–1.07], but no prospective association between neighborhood disadvantage and alcohol quantity × frequency at wave 5 (MR = 1.00, 95% CI = 0.97–1.02).

#### Cross‐sectional discordant twin/sibling models

In the base model, the within‐pair association between neighborhood disadvantage and alcohol quantity × frequency was not statistically significant, and the between‐pair association was qualified by a significant interaction with sex, wherein the association was significantly larger among females than among males (Figure 1c; Table 3). Each additional between‐pair decile in neighborhood disadvantage was associated with a significant 10% increase in the number of drinks consumed in the past month among women, and a non‐significant increase among men (Figure 1c; Table 3).

In the full model, the within‐pair association between neighborhood disadvantage and alcohol quantity × frequency became significant, indicating that none of the individual‐level covariates explained this neighborhood‐level effect. The between‐pair association between neighborhood disadvantage and alcohol use among women in the full model simultaneously including all the individual‐, family‐ and neighborhood‐level covariates was reduced, but remained statistically significant (Figure 1c; Table 3; for full results for females, see Table S5). Prior to the final model fitting, a control variable and several interactions were evaluated, and as they were not associated with alcohol quantity × frequency, they were not included in the models; see Table 3 and Appendix S1.

#### Prospective discordant twin/sibling models

Neighborhood disadvantage at wave 4 did not significantly predict alcohol quantity × frequency at wave 5 (Figure 1d; see Table 4).

## DISCUSSION

An assumption of most neighborhood alcohol research is that neighborhood characteristics represent community‐level risk factors that affect an individual's use of alcohol [4]. But it is also plausible that these might reflect effects of the individual [31] or the family of origin [7] on both the neighborhood of residence and alcohol use and misuse [32]. Because we cannot randomly assign individuals to different levels of neighborhood advantage [33] we can turn to a counterfactual model [34, 35], such as the discordant twin/sibling design [36]. Such a quasi‐experimental approach can adjudicate between alternate explanations for the association between neighborhood disadvantage and alcohol use and misuse.

In a national sample of mid‐adult twins and full siblings from the Add Health study, the cross‐sectional and prospective relations between two measures of alcohol use and one measure of alcohol misuse and neighborhood socio‐economic disadvantage were dissected into within‐pair and between‐pair effects; the results varied for the three alcohol outcomes. Neighborhood disadvantage was negatively, positively and not significantly associated with any alcohol use, quantity × frequency of use and binge drinking, respectively.

### Cross‐sectional neighborhood associations with alcohol outcomes

There was no evidence for a potentially causal influence of the neighborhood environment on alcohol use occurrence in the past year. That is, the twin or sibling who resided in a more disadvantaged neighborhood was no more or less likely to have consumed alcohol in the past year than his or her co‐twin or co‐sibling residing in a less disadvantaged neighborhood. However, there was evidence for family‐level confounding of the contemporaneous associations between neighborhood and any alcohol use related to characteristics of the twins or siblings who, by choice or circumstance, resided in more or less disadvantaged neighborhoods. For each additional between‐pair decile in neighborhood disadvantage, twins or siblings from pairs who, on average, were living in more disadvantaged neighborhoods were at 8% *decreased* odds of drinking in the past year than twins or siblings from pairs living in less disadvantaged neighborhoods.

In contrast, there was evidence of a potentially causal influence of the neighborhood environment on the amount of alcohol consumed in the past month. The twin or sibling who resided in a more disadvantaged neighborhood consumed 4% *more* drinks than his or her co‐twin or co‐sibling residing in a less disadvantaged neighborhood. There was also evidence for family‐level confounding of the association between neighborhood disadvantage and alcohol quantity × frequency among females but not among males, wherein females from twin or sibling pairs who, on average, were living in more disadvantaged neighborhoods reported consuming 9% more drinks in the past month than the females living in less disadvantaged neighborhoods.

### Prospective neighborhood associations with alcohol outcomes

The results of prospective analyses predicting the occurrence of any alcohol use or alcohol quantity × frequency nearly a decade after exposure to neighborhood disadvantage also differed for the two alcohol outcomes. For the occurrence of any alcohol use, the between‐pair association was as large in the prospective model as in the cross‐sectional model, and supplementary analyses of the individual‐level effects of wave 4 neighborhood disadvantage on any alcohol use across five waves of the Add Health study revealed that they were roughly equivalent. In contrast, between‐pair alcohol quantity × frequency (among women but not men) was associated only with contemporaneous neighborhood disadvantage, and the individual‐level effects across five waves of the Add Health study (Table S1) were also specific to contemporaneous neighborhood disadvantage at wave 4.

### Neighborhood disadvantage and alcohol use occurrence versus quantity consumed

Taken together, these results suggest that the between‐pair relations between neighborhood disadvantage and whether one drinks (any alcohol use), and among those who drink, how much is consumed (alcohol quantity × frequency), may reflect distinct processes. The negative between‐pair association between neighborhood disadvantage and any alcohol use may be associated with relatively stable differences between families. In the present study, the relation was reduced after accounting for the neighborhood‐ and family‐level factors of rural residence, race/ethnicity and SES (household income and educational attainment). This is consistent with the epidemiologic literature demonstrating that alcohol use is more prevalent among those with higher household incomes and greater educational attainment [37, 38], and that these are also the characteristics of the residents of less disadvantaged neighborhoods [39, 40]. This may be through differential selection by families into neighborhoods [7]. The positive between‐pair association between neighborhood disadvantage and alcohol quantity × frequency may be related to more transitory between‐family differences that are especially relevant to women. A handful of studies have found that women are more vulnerable than men to the effects of neighborhood disadvantage or disorder on alcohol use or misuse [41, 42, 43]. However, none of these studies controlled for genetic and environmental confounding of the relation. In the present study, the between‐pair association among women was slightly reduced by the inclusion of family‐ and individual‐level covariates. In particular, the personality trait of extraversion was associated with alcohol quantity × frequency and has also been associated with (un)willingness to relocate to a new neighborhood [44]. This is especially relevant to women because they are less willing to relocate than are men and this may be a transitory effect because they may be especially unwilling to relocate when they have young children [45]. Although speculative and in need of empirical verification, we posit that the between‐pair association between neighborhood disadvantage and alcohol quantity × frequency among women in their peak childbearing years [46] may be explained by familial factors that are associated with alcohol use [47] and with remaining in a disadvantaged neighborhood by choice and circumstance.

### Limitations

This study is not without limitations. First, the focus on socio‐economic disadvantage omitted some neighborhood characteristics that may be associated with drinking in mid‐adulthood (e.g. [48]). Second, studies focusing on general health [49] and mental health [50] have found that early‐life neighborhoods may be more important than the current neighborhood context, and this proved to be the case with respect to binge drinking in the present study (Table S1). Mid‐adult neighborhood at wave 4 was chosen because the twins or siblings would all have reached adulthood, been more likely to have moved from their shared childhood home and therefore more likely to be discordant for level of disadvantage in their neighborhood. Third, the 9 years between waves 4 and 5 may have been an overly stringent test of prospective prediction.

## CONCLUSION

Two previous discordant twin studies focused on alcohol misuse [15, 16]; the present study was novel in its focus on two complementary measures of alcohol use as well as a measure of misuse (binge drinking). The results suggested that the relations between neighborhood disadvantage and whether one drinks and the amount consumed may reflect distinct processes. There was evidence for a potentially causal effect of neighborhood disadvantage on the amount of alcohol consumed, but not whether one drinks or episodes of binge drinking. Neighborhood disadvantage associations were more consistently explained by between‐family confounding factors, that is, genetic and environmental factors related to neighborhood disadvantage on the one hand, and whether one drinks and the amount consumed on the other. This may be due to differential selection by families into neighborhoods.

## AUTHOR CONTRIBUTIONS

**Wendy S. Slutske:** Conceptualization; data curation; formal analysis; methodology; investigation; funding acquisition; writing—original draft; writing—review and editing; visualization. **Timothy B. Baker:** Supervision; writing—review and editing; resources. **Thomas M. Piasecki:** Visualization; writing—review and editing; formal analysis.

## DECLARATION OF INTERESTS

None.

## Supporting information


**Appendix S1.** Results of evaluations of an extra control variable and interactions for inclusion in multi‐level models.
**Table S1.** Neighborhood disadvantage sensitivity analyses.
**Table S2.** Results from cross‐sectional multi‐level discordant twin/sibling models predicting any past‐year drinking at wave 4 from neighborhood disadvantage at wave 4 before and after accounting for Wave 3 past‐year drinking.
**Table S3.** Results from prospective multi‐level discordant twin/sibling models predicting any past‐year drinking at wave 5 from neighborhood disadvantage at wave 4 before and after accounting for wave 4 past‐year drinking.
**Table S4.** Results from prospective multi‐level discordant twin/sibling models predicting any past‐year drinking at wave 5 from neighborhood disadvantage at wave 4 before and after accounting for wave 5 neighborhood disadvantage of the individual.
**Table S5.** Results from cross‐sectional multi‐level discordant twin/sibling models predicting past month quantity × frequency of alcohol use at wave 4 from neighborhood disadvantage at wave 4 among women.

## Data Availability

The data used in this study are available through contractual agreement with the National Longitudinal Study of Adolescent to Adult Health. More information is available at https://addhealth.cpc.unc.edu/data/restricted-use-data-sets/

## Footnotes

^1^
The term potentially causal is used throughout this article because causality cannot be definitively established using observational data. For example, in the discordant twin/sibling design, the underlying reason for discordance can never be known with certainty, thereby making it impossible to rule out alternative, non‐causal explanations.

^2^
The Add Health sample is nationally representative, but the twin/sibling subsample may not be. At wave 1, additional adolescents were selected outside of the sampling frame as part of the genetic sample. This was done to ensure that the sample size of genetically related individuals was large enough for specialized genetic analyses. As these adolescents were selected outside of the sampling frame, sampling weights could not be constructed.

^3^
A census tract is a small, relatively permanent geographic entity delineated by the United States Census Bureau, usually with a population size of between 1200 and 8000 people.

^4^
The five indicators were all significantly correlated with each other and loaded onto a single common factor.

^5^
The interaction between zygosity and the within‐pair effect provides a test of between‐family confounding. This is based on the level of control provided by discordant MZ and DZ/FS pairs. All three sibling types equally control for shared environmental confounding. MZ twins control for all genetic confounding, whereas DZ/FS control for half of the genetic confounding. An association between an exposure and outcome would be completely due to confounding if the effect among discordant DZ/FS pairs was less than the individual‐level effect and the effect among discordant MZ twins was less than that among DZ/FS pairs and did not differ from zero.

6 As Add Health used 2010 census geographic boundaries to create census tract, county and state identifiers that were longitudinally consistent across all five study waves, we were able to track consistencies and changes in census tract across waves.
