# Supplementary material for: A cross‐sectional and prospective examination of alcohol use and misuse among adult twin and sibling pairs discordant for neighborhood socio‐economic disadvantage
Source: Addiction. 2026 Jun 29;121(10):2713–24. doi: 10.1111/add.70533 (PMC13578849; doi:10.1111/add.70533)
Supplement: Supplementary file 1 [file ADD-121-2713-s001.docx]

**Supplemental Materials for**

A Cross-sectional and Prospective Examination of Alcohol Use and Misuse among Adult Twin and Sibling Pairs Discordant for Neighborhood Socioeconomic Disadvantage

Wendy S. Slutske, Timothy B. Baker, and Thomas M. Piasecki

**Contents**

1) Table S1. Neighborhood disadvantage sensitivity analyses.

2) Text S1. Results of evaluations of an extra control variable and interactions for inclusion in multilevel models.

3) Table S2. Results from cross-sectional multilevel discordant twin/sibling models predicting any past year drinking at Wave 4 from neighborhood disadvantage at Wave 4 before and after accounting for Wave 3 past year drinking.

4) Table S3. Results from prospective multilevel discordant twin/sibling models predicting any past year drinking at Wave 5 from neighborhood disadvantage at Wave 4 before and after accounting for Wave 4 past year drinking.

5) Table S4. Results from prospective multilevel discordant twin/sibling models predicting any past year drinking at Wave 5 from neighborhood disadvantage at Wave 4 before and after accounting for Wave 5 neighborhood disadvantage of the individual

6) Table S5. Results from cross-sectional multilevel discordant twin/sibling models predicting past month quantity x frequency of alcohol use at Wave 4 from neighborhood disadvantage at Wave 4 among women.

| **Table S1.** Neighborhood disadvantage sensitivity analyses. | | | | | | |
| --- | --- | --- | --- | --- | --- | --- |
| Panel A. Correlations between neighborhood socioeconomic disadvantage and past-month or past-year alcohol use and misuse as a function of wave/age period and time lag. | | | | | | |
|  | Alcohol Measure | | | | | |
|  | Any use^a^ | | Quantity-frequency^b^ | | Binge drinking^a^ | |
| Neighborhood disadvantage measure | Wave 4 | Wave 5 | Wave 4 | Wave 5 | Wave 4 | Wave 5 |
| Wave 1 (1994-1995) | **-0.19** | **-0.11** | -0.03 | -0.08 | **-0.15** | -**0.11** |
| Wave 2 (1996) | **-0.18** | **-0.06** | -0.02 | -0.07 | **-0.14** | -0.09 |
| Wave 3 (2001-2002) | **-0.13** | **-0.11** | 0.07 | -0.02 | -0.07 | -0.07 |
| Wave 4 (2008-2009) | **-0.15** | **-0.12** | **0.11** | -0.01 | -0.04 | -0.05 |
| Wave 5 (2016-2018) | **-0.16** | **-0.09** | 0.07 | -0.03 | -0.03 | -0.05 |
| Note: ^a^ past year, ^b^ past month  Binge drinking was log-transformed.  Correlations in bold are statistically significant at *p* < .0017 (.05/30).  Shaded row is the focus of the discordant twin/sibling analyses in the present study. | | | | | | |

| Panel B: Intercorrelations between measures of neighborhood disadvantage across waves | | | | | |
| --- | --- | --- | --- | --- | --- |
|  | Wave 1 | Wave 2 | Wave 3 | Wave 4 | Wave 5 |
| Wave 1 | --- |  |  |  |  |
| Wave 2 | **0.97** | --- |  |  |  |
| Wave 3 | **0.56** | **0.57** | --- |  |  |
| Wave 4 | **0.42** | **0.42** | **0.42** | --- |  |
| Wave 5 | **0.37** | **0.38** | **0.36** | **0.44** | --- |
| Note: Correlations in bold are statistically significant at *p* < .0001.  For all five waves, neighborhood disadvantage was a composite based on census-tract-level indicators extracted from the most recent census. The same five indicators were used for all five waves. At all five waves, the indicators were significantly correlated with each other and loaded onto a single common factor. | | | | | |

**Text S1.** Results of evaluations of an extra control variable and interactions for inclusion in multilevel models.

**Any Alcohol Use**

The mean age difference for non-twin full siblings was 2.28 (SD 1.16) years. Sensitivity analyses compared the correlations in full siblings (FS) and in dizygotic (DZ) and monozygotic (MZ) twins regarding any past year alcohol use (FS: *r* = 0.36; DZ: *r* = 0.38; MZ: *r* = 0.53) and neighborhood disadvantage (FS: *r* = 0.38; DZ: *r* = 0.39; MZ: *r* = 0.39). These correlations suggest that variation in any alcohol use and neighborhood disadvantage were both explained by familial factors (genetic or shared environmental factors) and unique environment. The similar levels of association for FS and DZ twins coupled with the stronger association among MZ twins for past year alcohol use suggests that genetic influences contributed to alcohol use, whereas the similar levels of association for FS, DZ, and MZ twins for neighborhood disadvantage suggests that differences in levels of neighborhood disadvantage were primarily due to shared (and unique) environmental influences.

The main effects for same-sex sibling (*p* = .31) and zygosity (*p* = .14) were not statistically significant. The zygosity x within-pair disadvantage interaction was significant in the base model (OR = 0.85, 95% CI = 0.72, 0.99, *p* = .04) but not in the full model (OR = 0.85, 95% CI = 0.72, 1.01, *p* = .06). None of the interactions between sex (within: *p* = 0.21; between: *p* = 0.89) and race (within: *p* = 0.59; between: *p* = 0.93) and within-pair and between-pair neighborhood disadvantage effects was significant. The non-significant predictors (same-sex sibling and sex and race x neighborhood interactions) were not included in models, but the significant zygosity x within-pair disadvantage interaction (along with the zygosity main effect) was.

**Alcohol Quantity x Frequency**

Sensitivity analyses compared the correlations in FS and in DZ and MZ twins regarding past month alcohol quantity-frequency (FS: *r* = 0.24; DZ: *r* = 0.28; MZ: *r* = 0.34). These correlations suggest that variation in alcohol quantity-frequency were explained by familial factors (genetic or shared environmental factors) and unique environment. The similar levels of association for FS and DZ twins coupled with the stronger association among MZ twins for alcohol quantity-frequency suggests that genetic influences contributed to differences in alcohol quantity-frequency.

The main effects for same-sex sibling (*p* = .81) and zygosity (*p* = .13) were not statistically significant. The interactions between zygosity (within: p = 0.22) and race (within: *p* = 0.07; between: *p* = 0.12) with within-pair and between-pair neighborhood disadvantage effects were also not significant, but there was a significant sex interaction with neighborhood disadvantage in the base model (*p* = .02) and the full model (between: *p* = .02), wherein the between-pair neighborhood disadvantage effect was significantly larger among females (*p* = .0001) than among males (*p* = .12) in the base model and in the full model (females: *p* = .0001, males: *p* = 0.74). The non-significant predictors (same-sex sibling and zygosity, and zygosity and race x neighborhood interactions) were not included in final models, but the significant sex x between-pair disadvantage interaction was.

| **Table S2.** Results from cross-sectional multilevel discordant twin/sibling models predicting any past year drinking at Wave 4 from neighborhood disadvantage at Wave 4 before and after accounting for Wave 3 past year drinking. | | | | |
| --- | --- | --- | --- | --- |
|  | **Base model** | | **Base model with**  **Wave 3 drinking** | |
| **Predictor** | **OR** | **95% CI** | **OR** | **95% CI** |
| Within-pair ND | 0.96 | 0.90, 1.01 | 0.99 | 0.93, 1.06 |
| Between-pair ND | **0.87** | **0.82, 0.91** | **0.91** | **0.86, 0.95** |
| Female sex | **0.52** | **0.43, 0.64** | **0.56** | **0.45, 0.69** |
| Age | **0.92** | **0.87, 0.97** | **0.89** | **0.84, 0.95** |
| Race/ethnicity |  |  |  |  |
| White (reference) | --- | --- | --- | --- |
| Black | **0.51** | **0.38, 0.67** | **0.70** | **0.52, 0.93** |
| Other | **0.61** | **0.47, 0.79** | **0.75** | **0.57, 0.98** |
| Wave 3 drinking |  |  | **5.71** | **4.47, 7.29** |
|  |  |  |  |  |
| Note: Estimates in bold are significant at *p* < 0.05.  ND = neighborhood disadvantage | | | | |

| **Table S3.** Results from prospective multilevel discordant twin/sibling models predicting any past year drinking at Wave 5 from neighborhood disadvantage at Wave 4 before and after accounting for Wave 4 past year drinking. | | | | |
| --- | --- | --- | --- | --- |
|  | **Base model** | | **Base model with**  **Wave 4 drinking** | |
| **Predictor** | **OR** | **95% CI** | **OR** | **95% CI** |
| Within-pair ND | 0.93 | 0.85, 1.01 | 0.95 | 0.86, 1.04 |
| Between-pair ND | **0.85** | **0.80, 0.91** | **0.90** | **0.84, 0.96** |
| Female sex | 0.96 | 0.72, 1.29 | 1.31 | 0.97, 1.75 |
| Age | 0.96 | 0.88, 1.04 | 0.99 | 0.91, 1.07 |
| Race/ethnicity |  |  |  |  |
| White (reference) | --- | --- | --- | --- |
| Black | 0.68 | 0.46, 1.01 | 0.98 | 0.67, 1.45 |
| Other | 0.89 | 0.61, 1.31 | 1.13 | 0.77, 1.65 |
| Wave 4 drinking |  |  | **9.69** | **6.70, 14.00** |
|  |  |  |  |  |
| Note: Estimates in bold are significant at *p* < 0.05.  ND = neighborhood disadvantage | | | | |

| **Table S4.** Results from prospective multilevel discordant twin/sibling models predicting any past year drinking at Wave 5 from neighborhood disadvantage at Wave 4 before and after accounting for Wave 5 neighborhood disadvantage of the individual | | | | | |
| --- | --- | --- | --- | --- | --- |
|  | **Base model** | | **Base model**  **with Wave 5 ND** | | |
| **Predictor** | **OR** | **95% CI** | **OR** | **95% CI** | |
| Within-pair ND | 0.93 | 0.85, 1.01 | 0.94 | 0.86, 1.03 | |
| Between-pair ND | **0.85** | **0.80, 0.91** | **0.89** | **0.83, 0.96** | |
| Female sex | 0.96 | 0.72, 1.29 | 0.95 | 0.70, 1.27 | |
| Age | 0.96 | 0.88, 1.04 | 0.94 | 0.87, 1.03 | |
| Race/ethnicity |  |  |  |  | |
| White (reference) | --- | --- | --- | --- | |
| Black | 0.68 | 0.46, 1.01 | **0.66** | **0.44, 0.99** | |
| Other | 0.89 | 0.61, 1.31 | 0.90 | 0.61, 1.33 | |
| Wave 5 ND |  |  | 0.96 | 0.90, 1.01 | |
|  |  |  |  |  | |
| Note: Estimates in bold are significant at *p* < 0.05.  ND = neighborhood disadvantage  Wave 5 neighborhood disadvantage was a composite based on census-tract-level indicators extracted from the 2014-2018 census. The five indicators were the same as used for Wave 4 neighborhood disadvantage. Similar to Wave 4, the indicators were also significantly correlated with each other and loaded onto a single common factor. | | | | |  |

| **Table S5.** Results from cross-sectional multilevel discordant twin/sibling models predicting past month quantity x frequency of alcohol use at Wave 4 from neighborhood disadvantage at Wave 4 **among women**. | | | | |
| --- | --- | --- | --- | --- |
|  | **Past month quantity-frequency of use** | | | |
|  | **Base model** | | **Fully adjusted model** | |
| **Predictor** | **MR** | **95% CI** | **MR** | **95% CI** |
| Within-pair ND | 1.04 | 0.99, 1.09 | 1.03 | 0.98, 1.08 |
| Between-pair ND | **1.10** | **1.05, 1.16** | **1.09** | **1.03, 1.15** |
| Age | 0.95 | 0.89, 1.00 | 0.96 | 0.91, 1.02 |
| Race/ethnicity |  |  |  |  |
| White (reference) | --- | --- | --- | --- |
| Black | 0.75 | 0.56, 1.01 | 0.80 | 0.59, 1.08 |
| Other | 1.22 | 0.94, 1.58 | 1.20 | 0.92, 1.58 |
| Rural |  |  | 0.78 | 0.50, 1.21 |
| Parental SES |  |  | **1.12** | **1.01, 1.23** |
| Household income |  |  | 0.99 | 0.97, 1.02 |
| Educ. attainment |  |  | 0.97 | 0.89, 1.06 |
| Personality |  |  |  |  |
| Neuroticism |  |  | **1.10** | **1.00, 1.20** |
| Extraversion |  |  | **1.29** | **1.17, 1.42** |
| Openness |  |  | 1.02 | 0.92, 1.13 |
| Agreeableness |  |  | **0.82** | **0.73, 0.93** |
| Conscientiousness |  |  | 0.92 | 0.84, 1.01 |
| Note: Estimates in bold are significant at *p* < 0.05, MR = mean ratio, CI = confidence interval, ND = neighborhood disadvantage | | | | |
